# Micro-Vesiculation and Disease, London, 13–14 September 2012

**DOI:** 10.3402/jev.v1i0.19768

**Published:** 2012-10-30

**Authors:** Raymond M. Schiffelers

**Affiliations:** Laboratory of Clinical Chemistry and Hematology, Division of Laboratories and Pharmacy, University Medical Center Utrecht, Utrecht, The Netherlands

The Biochemical Society (London, UK) held a “Focused meeting” on “Micro-Vesiculation and Disease” on 13–14 September 2012 within the London Metropolitan University, London, UK. Focused meetings are dedicated to emerging life science topics. This meeting was organised by Jameel Inal, Una Fairbrother, Sheelagh Heugh and Annie Bligh and supported by Niamh Mangan and Caron Barter. The organisers aimed to bridge the findings on extracellular vesicles at the cellular level with organismal research on therapy and pathophysiology. The meeting was centred around a selected set of longer talks touching upon different subjects and specifically allowed longer times for discussion. Finally, speakers were asked to write short review articles based on their talks, to be published in a special issue of *Biochemical Society Transactions*.

Note that this short report summarises the author's understanding of the different presentations. The author apologises in advance to the presenters for any unwilling inaccuracy.

*Rémy Sadoul* (Grenoble, France) kicked off with a presentation on the function of neuronal exosomes. Within brain research, signal transduction has conventionally been interpreted as local changes in transcription, translation and post-translational modifications driving the generation of new circuits that connect neurons. The likelihood that exosomes can function as a liaison between neurons offers new possibilities. The action of exosomes appears to be in the synapses, where receptors reside on spines protruding from dendritic shafts. Multi-vesicular bodies can be detected in the vicinity of these synapses. The work of Sadoul et al. suggests that rat embryo cortical neurons secrete exosomes, which contain specific sets of cytosolic and membrane proteins (e.g. enriched in GluR 2/3 and depleted of Tau and PSD95) as well as miRNAs. mRNAs were not recovered. Secretion of exosomes was strongly up-regulated by calcium influx regulated by glutamatergic activity, as evidenced by bicuculline inhibition of inhibitory signals, suggesting their role in normal synaptic signalling. Work on tetanus toxin, a toxin that resides in multi-vesicular bodies within synapses, was shown to be transported trans-synaptically to other neurons. This offers a possible route of transmission for pathogenic proteins in diseases like Parkinson's and Alzheimer's.

*Theresa L. Whiteside* (Pittsburgh, PA) spoke about the immune suppressive activity of tumour-derived vesicles. These vesicles can be isolated from body fluids of patients with cancer. They contain various immunologically active molecules such as FasL, MHC class I and II, and tumour-associated antigens. Nevertheless, the expression of these molecules is not uniform and even a panel of three ovarian cancer cell lines, cultured in the same way, differ substantially in their protein “fingerprint”. Results show that tumour-associated antigens on the surface of these vesicles are not immunogenic but rather immunosuppressive, FasL was shown to drive anti-tumour T cells into apoptosis and vesicles could induce the formation of immunosuppressive regulatory T-cells. This indicates a broad repression of immune function in the tumour at many different levels. Preliminary data on serum vesicles from melanoma patients indicated that melanoma grade correlates with the amount of vesicles and that treatment with interferon-α reduces these levels compared to vesicle concentrations before treatment indicating that vesicles may have diagnostic potential.

*Marcel Ramirez* (Rio de Janeiro, Brazil) discussed the role of vesicles in the communication between species, that is, parasites and hosts. In their studies, they focus on *Trypanosoma cruzi* (the cause for Chagas’ disease), which, when it is successful in infection, can evade the complement system. Apart from molecular inhibitors, a role seems available for extracellular vesicles. It was shown that bot parasites and monocytes that were in contact with *T*. *cruzi* produced micro-vesicles. These vesicles inhibited complement-mediated lysis of parasites and increased infectivity. Interestingly, fusion between vesicles from parasites and monocytes occurred. The role of these fused vesicles is under investigation as is the role of micro-vesicles in the infectivity of other parasites, such as *Giardia spp*.

*Amy Buck* (Edinburgh, Scotland) focused on a different parasite, the nematode *Heligmosomoides polygyrus*. Her work centres on the ability of parasites to affect regulatory RNAs and influence gene expression in the host. Historically, this has been the field of virus–host interaction, but her work also indicates that regulatory RNAs are important in nematode–host interactions. In particular, miRNAs are key regulators of gene expression in the host in this respect. Nematodes have many different molecules to combat immune recognition and destruction and also produce micro-vesicles. miRNA screening revealed 658 unique miRNA precursors, of which 78 are abundantly expressed. The vesicles appear enriched in miRNAs that are homologous to mouse miRNAs, importantly miRNAs that have also been shown to regulate immune responses, for example, interleukin-6.

*Jameel Inal* (*London, United Kingdom)* started with discussing methods to collect and quantitate vesicles and the average concentrations obtained in samples. They also studied the role of vesicles in *T*. *cruzi* infection. They are particularly interested in the interaction of the infectious metacyclic trypomastigote form, when it is infecting host cells. During this interaction, the parasite binds integrins and lipid rafts and activates stretch-activated channels, leading to calcium influx and vesiculation. The local changes in the membrane after vesiculation led to localisation of lysosomes in the vicinity. This induction of vesiculation with accompanying local membrane changes appears to be used by the parasite to increase infectivity as inhibitors of vesiculation, and like calpeptin and gadolinium chloride, decrease parasite infection. At present, the studies are expanded to include *Salmonella typhimurium*, a facultative intracellular bacterium, which also induces micro-vesicle formation. Interestingly, they have even expanded their studies to viruses where the vesiculation responses also seem to aid the virus in spreading rather than inhibiting it. In studies with coxsackievirus B1, the apoptotic response of cells following viral infection is usually interpreted as a process that limits viral transmission to other cells. However, Inal et al. show that coxsackievirus infection leads to vesiculation and apoptosis induction, which is inhibited by calpeptin-mediated inhibition of vesicle shedding. Also, calpain siRNA limits micro-vesicle release, vesicles that were shown to contain virus in addition to death molecules such as caspase 3 that could activate the apoptosis program in recipient cells.

*Clotilde Thery* (Paris, France) spoke of the possible dual role of exosomes. In principle, they could be used for antigen transfer to dendritic cells, but there is also evidence that shows that they suppress anti-tumour effects. However, all of these studies have been performed in vitro with purified exosomes, which could be different from studies carried out in vivo. To be able to study this, Clotilde's lab worked on tools to inhibit exosome secretion. In a screening with an shRNA library, Rab proteins (2, 5, 9, 27a and b) in particular were shown to inhibit exosome secretion. Rab 27 appeared specifically important for localisation at the plasma membrane and release, at least in HeLa cells. In mouse tumour cells, the situation was very different. B16 cells contained only Rab27a. In 4T1, expression of 27a and b was equal but only 27a reduced exosomes. However, this depends on the exosome marker you use to detect them. Some markers were left unchanged while others were reduced. This could indicate that 27a silencing reduces release of a subset of vesicles. It appears that classical 100,000×g pellets contain different types of vesicles, whose release is dependent on different pathways. Finally, studies were expanded to in vivo experiments, also yielding different results for different tumours. In TSA tumours, Rab 27a had no effect, but in 4T1 tumours it reduced tumour growth, which was postulated to be due to inhibition of neutrophil influx in this model. Reinjection of exosome restores influx as well as tumour growth.

*Monika Baj-Krzyworzeka* (Cracow, Poland) discussed the role of monocytes that infiltrate tumours, become M2 polarised and, finally, converted to tumour-associated macrophages. It appears that vesicles shed from the tumour cells dictate the polarisation and conversion of monocytes. Results were presented on the vesicles of a variety of tumour cell lines in culture. The vesicles were surprisingly large, with a size range of 0.5–2 µm. The vesicles showed a heterogeneous composition and, in part, reflected the cell of origin. Following incubation with micro-vesicles, monocytes change morphology, receive tumour-associated antigens, and secrete specific cytokines and chemokines. It was shown that the vesicles interacted differently with different sub-populations of monocytes that could be distinguished by their CD14 and CD16 levels.

*Mattias Belting* (Lund, Sweden) described work on malignant brain tumours that typically have regions of hypoxia and hyper-coagulation. Their work shows that hypoxic cancer cells release vesicles that are decorated with tissue factor. The contents of these hypoxia-induced vesicles closely resemble the hypoxic signature of the cells that they are derived from. The tissue factor is transferred to protease-activated receptor-2, a receptor induced on hypoxic endothelial cells. This binding can activate the cells in a paracrine manner. Apart from cell activation, the hypoxic vesicles induce sprouting of endothelium. These pro-angiogenic activities are also maintained in vivo. Glioblastoma-bearing mice that receive an injection with micro-vesicles on day 1 show enhanced tumour growth, increased tumour weight and high micro-vessel density over the following month compared to controls. Vesicles seem to be an important stimulant of hypoxia-mediated tumour growth.

*Stephen J. Gould* (Baltimore, MD) focused on the location of budding of exosomes and other vesicles. His results, primarily in non-adhering T-cells show a different picture than the generally assumed membrane source for exosomes. Although people generally assume that exosomes are budding into multi-vesicular bodies, and subsequently get released at the cell membrane, Stephen's results show that in T-cells, the budding occurs primarily directly from the cell membrane. The signal that dictates whether proteins are incorporated into these vesicles appears to be oligomerisation. Artificial or natural induction of oligomerisation by antibodies that cross-link membrane proteins or by membrane anchors for naturally oligomeric cytoplasmic proteins ensures their localisation at sites on the cell membrane of active budding. In contrast, targeting proteins to the endosomal membrane by providing a 2xFYVE signal does not lead to budding, which seems to indicate that plasma membrane budding is the predominant form of exosome secretion in this cell type. Interestingly, the oligomerisation has strong parallels with the Gag-protein-driven oligomerisation during HIV budding, which indicates that these two pathways may have the same underlying principle. An intriguing finding was that the SP2 peptide in Gag forms a strong inhibitor of budding, which appears to be due to binding to an endogenous regulator of vesicle secretion.

*Eva Ogorevc* (Ljubljana, Slovenia) began by illustrating the difficulties of reproducible isolation of vesicles from blood of patients. She showed data of three departments within the same institute that isolated vesicles using the same protocol. Temperature and shear stress were crucial parameters to control vesicle yield. They used the improved protocol to quantify vesicles in blood during imatinib treatment in patients with gastro-intestinal stromal tumours. It was shown that there was a surge in vesicle concentrations immediately following the start of therapy, which rapidly fell back to pre-treatment levels and remained constant over a 32-month follow-up period.

*Samireh Jorfi* (London, United Kingdom) talked about the implications of vesicles in drug resistance of cancer cells. In the classical view, pumps, such as P-glycoprotein, mediate drug efflux but vesicles could be another efflux mechanism possibly for different classes of drugs. By pre-treating cancer cells in vitro with calpeptin, vesicle release was inhibited and sensitivity of cancer cells to methotrexate and docetaxel was restored. This increased sensitivity also was maintained in vivo.

*Giovanni Camussi* (Torino, Italy) began with discussing the possible uses of stem cells and the often surprisingly positive outcomes of clinical trials. These better-than-expected results sparked an interest in factors other than repopulation that could improve outcome. In the paracrine theory, the soluble factors and also vesicles that stem cells release are primarily responsible for their beneficial effects. He described a mechanism whereby stem cells could reprogram injured cells via vesicles with certain stem cell characteristics, and the injured cells could activate stem cells via their vesicles. In particular, miRNAs are shown to be largely responsible for pro-angiogenic effects of vesicles, in particular miR126 and miR296. Surprisingly, treating vesicles with RNAs eliminates the miRNAs and consequently strongly reduces the pro-angiogenic activity. As it is generally assumed that the RNAs are protected inside vesicles, either the incubation conditions are too harsh or the miRNAs are accessible on the outside of the vesicles

*Ray Schiffelers* (Utrecht, The Netherlands) spoke on erythrocyte vesicles. He showed that erythrocytes could shed vesicles upon cellular stress such as hyperthermia or oxidative stress. The resulting vesicles can be taken up by endothelium and cause up-regulation of a variety of iron processing enzymes and remarkable little toxicity. It appears that endothelial cells can act as professional erythrocyte-processing enzymes in this respect. When looking for erythrocyte vesicles in patient plasma, this is often obscured by their fast clearance. Ex vivo set-ups indicate, however, that they are readily formed but through their exposure of phosphatidylserine rapidly taken up by phagocytes and possibly endothelial cells.

*Sean Davidson* (London, UK) investigated the cardio-protective effects of vesicles after ischemia–reperfusion injury in rat hearts. In general, it is perceived that vesicles are detrimental in the circulation because, for example, they can contribute to thrombosis. They isolated vesicles from healthy rat plasma, recovering ~10^11^ vesicles per millilitre with an average size ~90 nm. These vesicles were shown to increase in vitro survival of cardiac cells and also to reduce infarct sizes in ex vivo perfused rat hearts.

The work of RMS on extracellular vesicles is funded through European Research Council Starting Grant 260627 – “Microvesicle-Inspired drug delivery systems”.

Raymond M. Schiffelers
Laboratory of Clinical Chemistry and Hematology,
Division of Laboratories and Pharmacy,
University Medical Center Utrecht, Utrecht,
The Netherlands

